# Generating and predicting high quality action plans to facilitate physical activity and fruit and vegetable consumption: results from an experimental arm of a randomised controlled trial

**DOI:** 10.1186/s12889-016-2975-3

**Published:** 2016-04-12

**Authors:** Dominique Alexandra Reinwand, Rik Crutzen, Vera Storm, Julian Wienert, Tim Kuhlmann, Hein de Vries, Sonia Lippke

**Affiliations:** Department of Psychology and Methods, Jacobs University Bremen, Campus Ring 1, Bremen, 28759 Germany; CAPHRI, Department of Health Promotion, Maastricht University, P. O. Box 616, Maastricht, 6200 MD The Netherlands; Institute for Social Medicine and Epidemiology, University of Lübeck, Ratzeburger Allee 160, 23562 Lübeck, Germany; Department of Psychology, University of Konstanz, 78457 Konstanz, Germany

**Keywords:** Physical activity, Fruit and vegetable consumption, Action plan, Plan quality, Instrumentality, Specificity

## Abstract

**Background:**

In order to improve the transition from an intention to a change in health behaviour, action planning is a frequently used behavioural change method. The quality of action plans in terms of instrumentality and specificity is important in terms of supporting a successful change in health behaviour. Until now, little has been known about the predictors of action plan generation and the predictors of high quality action plans and, therefore, the current study investigates these predictors.

**Method:**

A randomised controlled trial was conducted to improve physical activity (PA) and fruit and vegetable (FV) consumption using a web-based computer tailored intervention. During the 8-week intervention period, participants in the intervention arm (*n* = 346) were guided (step-by-step) to generate their own action plans to improve their health behaviours. Demographic characteristics, social cognitions, and health behaviour were assessed at baseline by means of self-reporting. Whether participants generated action plans was tracked by means of server registrations within two modules of the intervention.

**Results:**

The action planning component of the intervention regarding physical activity and fruit and vegetable consumption was used by 40.9 and 20.7 % of the participants, respectively. We found that participants who were physically active at baseline were less likely to generate action plans concerning physical activity. With regards to generating fruit and vegetable action plans, participants with a high risk perception and a strong intention to eat fruit and vegetables on a daily basis made more use of the action planning component for this behaviour. Finally, the large majority of the action plans for physical activity (96.6 %) and fruit and vegetable consumption (100 %) were instrumental and about half of the action plans were found to be highly specific (PA = 69.6 %/FV = 59.7 %). The specificity of the action plans is associated with having a relationship and low levels of negative outcome expectancies.

**Conclusion:**

Risk perception and intention are predictors of using the application of action planning. Increasing the motivation to change behaviour should be prioritised in interventions concerning changes in health behaviour before participants are asked to generate action plans. This would also make the intervention suitable for unmotivated people. For those participants who already perform the desired health behaviour prior to the intervention, action plans might be less relevant. Nevertheless, using a guided step-by-step approach to generate action plans resulted in highly instrumental and specific action plans and might be integrated into other interventions concerning changes in health behaviour.

**Trial Registration:**

Netherlands Trial Register: NTR 3706, ClinicalTrials.gov: NCT01909349.

## Background

Even highly motivated people can have problems in translating their intentions into a successful change in health behaviour [1]. Action planning has been identified as an important method to overcome this “intention-behaviour-gap” [2, 3] by improving self-regulatory skills [4]. Action plans (AP) specify precisely when, where, and how an intended health behaviour will be carried out [5–7]. When asking people to generate APs, such people should be encouraged to think about the context in which the desired behaviour will be performed. Due to these cues to action, such APs should work as a reminder to act (in terms of time and place) [8]; even when other self-regulatory skills and memory capacity are low, planning contributes to habit formation [9, 10].

Generating APs has been demonstrated to increase the translation from intention to behaviour for different health behaviours such as PA [11–14] and a healthy diet [15, 16]. The theoretical method of action planning is derived from several social-cognitive health theories such as the Health Action Process Approach (HAPA) [4, 17, 18]. It can be easily applied [19] and its application is understandable to users [8] and, hence, it is often applied to web-based interventions to encourage a change in health behaviour [8, 20].

The HAPA assumes that health behaviour is determined by social cognitions like risk perception, self-efficacy, and outcome expectancies as more distal determinants that influence intention as a more proximal determinant. Intention influences behaviour via action planning. HAPA assumes that action planning is determined by self-efficacy and intention [4]. It has been shown that people who have a high self-efficacy to perform a specific behaviour [21–23], have positive outcome expectations, have strong intentions [24, 25], are older, and are female [25] are more engaged in making APs. Furthermore, people with a low educational level have been found to use web-based health interventions less frequently than is recommended in terms of participating in the recommended modules [26]. Those in a relationship and those who are unemployed follow intervention recommendations more closely compared to singles and employed people [26]. Therefore, it might be possible that people with different educational levels, different relationship statuses, and different working conditions make different use of the intervention AP application.

While APs have been found to be a useful tool to improve a change in health behaviour, especially for fruit and vegetable (FV) consumption and physical activity (PA) [24, 27, 28], the quality of the APs might also have an influence on the change in health behaviour. The quality of APs can be described in terms of instrumentality and specificity [29]. Instrumental plans are goal-directed and describe an action that will result in the desired behaviour. For example, the desired behaviour is to be physically active and the AP focuses on using a bicycle to go to work. An AP would not be instrumental if the plan is not linked to the desired behaviour, such as eating less snacks. Furthermore, APs can vary in the degree of details provided (i.e. when, where, how, and with whom) [30] and studies have revealed that the more specific an AP, the more likely it is that the intended behaviour will be performed [13, 31–33].

Unlike social cognitions, personal characteristics of people generating an AP and the AP quality, has not been discussed in existent literature. However, it is important to understand if a practical application to generate APs is used equally between different subgroups (i.e., higher and lower educated).

The aim of this study is to evaluate the use of the intervention and the quality of the generated APs. Therefore, our first research question is: How are the modules from a web-based computer tailored intervention used and what are the predictors of intervention use? The second question is: Which predictors of generating APs for both behaviours can be identified? The third question is: What is the quality of the generated APs in terms of instrumentality and specificity? Finally, our last research question is: What are the predictors of AP specificity?

## Methods

### Ethical approval

The data analysis for the present paper was collected from a randomised controlled trial that tested the effectiveness of a web-based tailored intervention to facilitate PA and FV consumption. A study protocol about the study design and intervention has been published elsewhere [34]; in the following part, only the relevant details for the analyses in this paper will be described. The study received ethical approval in the Netherlands by the Medical Ethics Committee of Atrium Medical Centre Heerlen (METC number 12-N-124) and in Germany by The German Society for Psychology (DGPs; EK-A-SL 022013). All participants were asked to sign online informed consent, explaining the voluntariness and anonymity of their participation.

### Participant recruitment

Participants for this study were recruited in the Netherlands and Germany by advertising in hospitals, in newspapers, on social media networks, and by using research panels (Flycatcher in the Netherlands and Dr. Grieger & Cie in Germany). Participants aged 20 and older who were interested in reducing their cardiovascular risk by improving their PA and FV consumption were invited to participate.

### Study design and intervention content

Participants were allocated randomly by the computer software TailorBuilder [35] (a programme for web-based computer tailored interventions) into either the intervention group or the waiting list control group. We only made use of the information gained from the intervention group for the current study. At baseline, all participants filled in an online baseline questionnaire and, thereafter, participants in the intervention group gained access to the web-based intervention.

**Fig. 1:**
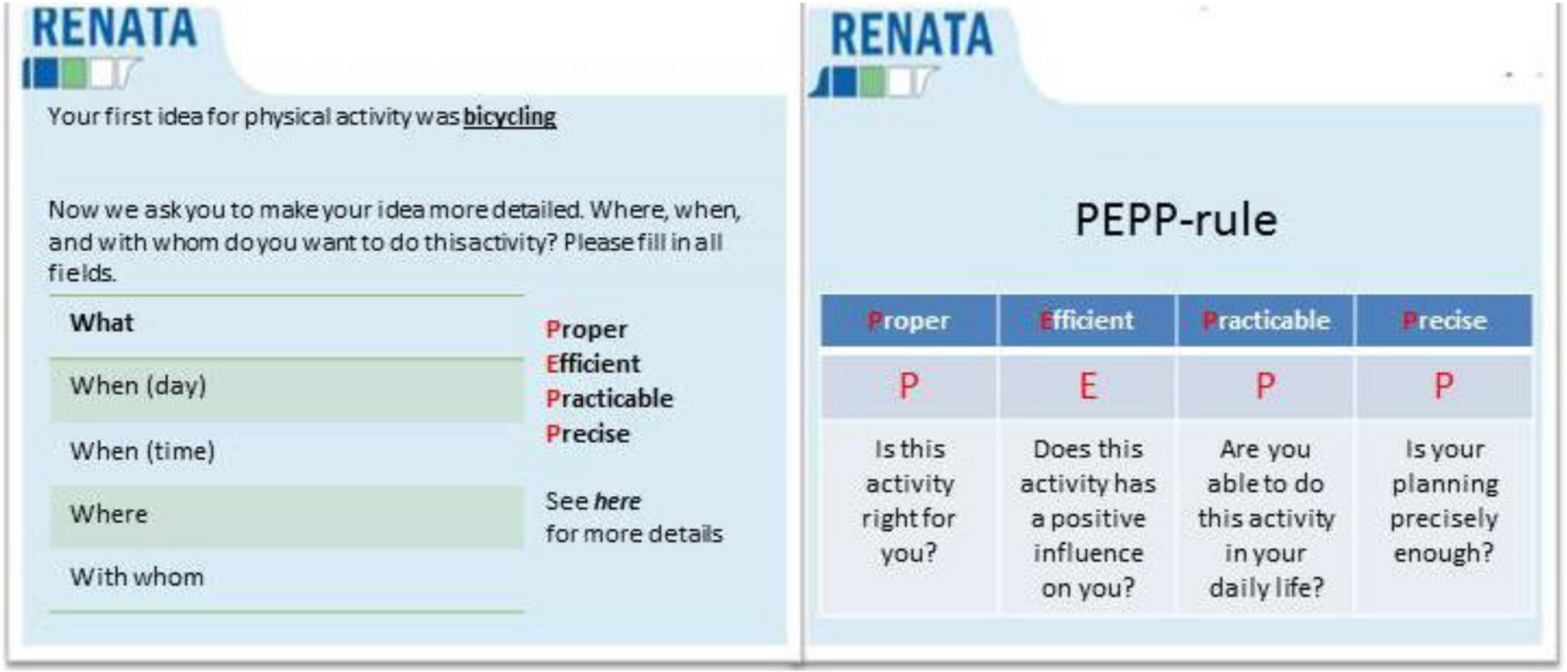
Example of how to generate APs within the intervention (PEPP-rule based on Fleig et al., [34])

The intervention consisted of an 8-week online programme in which participants were encouraged to log in once a week to participate in a specific module. The first four modules addressed PA and the last four modules focused on FV consumption. During the first module, participants were asked to generate a general goal with regards to PA (FV consumption was dealt with in the 5th module). In the second module, participants were guided to generate APs as precisely as possible; for which they were encouraged to generate “which day”, “at what time”, “how long”, “where”, and “with whom” they were planning to be physically active (or eating FVs during module six). Participants were asked to evaluate their own APs using the “PEPP-rule” (Fig. 1). PEPP means “Proper”, “Efficient”, “Practicable”, and “Precise” and this rule is used to avoid unrealistic planning [34]. In each module, participants received tailored feedback with regards to their health behaviours and planning progress. All participants were asked to formulate APs. Those who fulfilled the behavioural guidelines received the feedback that planning is a useful tool to maintain the desired behaviour in the long run. Participants who did not reach the behavioural guidelines received the feedback that planning would help to translate their intentions into behaviour even in difficult situations. Participants were able to reflect on their APs and adjust them if necessary. The data from the APs that participants generated during the two intervention modules has been used for this paper [36].

### Measures

The following *demographic information* was assessed: age, gender (1 = male, 2 = female), educational level (1 = low, 2 = middle, 3 = high), relationship (1 = single, 2 = partnership), and working situation (1 = unemployed, 2 = employed).

To assess health behaviour, the level of *PA* was measured using the short version of the International PA Questionnaire (IPAQ) [37, 38]. Assessing FV consumption during the past 7 days was done with the use of the Behavioral Risk Factor Surveillance System (BRFSS) questionnaire which asks participants to count the number of portions of fruit, vegetables, and salads they ate (on average) during a day [39].

The recommendation to be physically active at least 5 days in a week for at least 30 min per day [40] was used to categorize participants in terms of whether they fulfil the recommendation (2) or not (1). The same coding was used for FV consumption. Participants who ate (on average) five portions of FVs each day were classified as being compliant with the recommendation [41].

*Risk perception* with regards to cardiovascular diseases was measured in terms of five items, including: *“How likely is it that you will sometime in your life have: …” “… a high cholesterol level?”* or *“… a stroke*?” (Ω = 0.88, CI = 0.87-0.90). Possible responses ranged from 1 = totally disagree to 7 = totally agree.

*Outcome expectancies* were measured in terms of two positive items (*r* = 0.69) and two negative items (*r* = 0.34) concerning PA. Similarly, FV outcome expectancies were assessed in terms of two positive (*r* = 0.64) and two negative (*r* = 0.53) outcome expectancies. For example: “Being physically active for at least 30 min a day for at least 5 days a week will make me feel better”, “Eating 5 portions of FV a day will be good for my health” [42]. Items could be answered on a 7-point Likert scale (1 = totally disagree – 7 = totally agree).

*Self-efficacy* was assessed in terms of five items for PA (Ω =0.88, CI = 0.86–0.89) and five items for FVs (Ω = 0.91, CI = 0.90–0.92). Examples of such items include: *“I am certain that I can be physically active a minimum of 5 days a week for 30 min even it is difficult”, “I am certain that I can eat at least 5 portions of fruit and vegetables a day even if it is difficult”* (motivational) [43]. *“I am certain that I can be physically active permanently at a minimum of 5 days a week for 30 min …/”I am certain that I can permanently eat 5 portions of fruit and vegetable a day…” “… even if it takes a lot of time till I am used to do it”* (maintenance) [44], and *“I am certain that I can again be physically active a minimum of 5 days a week for 30 min/I am certain that I can again eat 5 portions of fruit and vegetables a day …” “even if I changed my concrete plans several times”* (recovery) [44]. Possible responses ranged from 1 = totally disagree to 7 = totally agree.

*Intention* to be physically active was assessed in terms of two single items: *“On 5 days a week for 30 min (or a minimum of 2.5 h per week), I have the intention to perform…”* 1: *“…intensive* physical activity*”,* 2 *“… moderate* physical activity*”.* Intention about FV consumption was assessed in terms of one item: “*I seriously intend to eat at least 5 portions of* fruit and vegetable *daily”* with possible responses ranging from totally agree (=7) to totally disagree (=1) [45].

To determine the AP quality in terms of specificity and instrumentality, information from the intervention itself was used. Whether participants generated a meaningful AP in module 2 (about PA) or in module 6 (about FV consumption) was coded as whether an AP was generated (=2) or not (=1). To assess the quality of the APs, we distinguished between instrumentality and specificity. We considered an AP to be *instrumental* when participants generated a goal-directed action (1). A plan was not instrumental (0) when the described action would not result in improving PA or FV consumption. Specificity was only defined in terms of instrumental APs. We categorised *specificity* into three categories: A plan is not specific (0) when participants had only generated what to do. Medium-specific (1) is defined as a plan that provided additional details about the time and day on which the action will be performed. A plan was considered highly specific (2) when it also described where and with whom the action would be done (participants were also allowed to comment that they wished to perform their behaviour alone) [31]. The APs were independently coded by two researchers. In 14 out of 438 cases, there was a discrepancy between the coding of the researchers, which was resolved by means of discussion.

### Statistical analysis

The data was analysed using SPSS software version 21 (IBM Corp, Armonk, NY, USA). Descriptive statistics were used to describe study sample characteristics. With regards to our first research question, a stepwise linear regression analysis was used to assess the predictors of intervention use whereby intervention use was defined in terms of the number of modules that participants completed (possible range: 0–8 modules).

In line with the HAPA model, variables were included in three steps: the first model contained only socio-demographic variables (age, gender, relationship, working situation, and educational level), the second model included (in addition to the first model) risk perception, outcome expectancies, self-efficacy, and whether participants’ behaviours are in line with the specific recommendation (yes = 2 no = 1). The final model also included intention (in addition to the first and second model).

Regarding research question two, a stepwise logistic regression analysis was used to assess predictors of action planning (yes = 2 or no = 1), by using the same three above mentioned models. One analysis was undertaken using action planning for PA as a dependent variable and one analysis was undertaken using action planning for FV consumption as a dependent variable.

In order to describe the quality of the generated APs, descriptive statistics were used.

Next, a stepwise linear regression analysis was undertaken to assess the predictors of making specific APs (research question four) for PA and for FV consumption, using the same three above mentioned models. When participants generated two plans for one of the behaviours, a mean score was calculated for the specificity of the plans. We excluded APs that were not classified as instrumental in the analysis such as: “undertake renovations”, “doing some arm-wrestling”, or “no idea” because they are irrelevant to this study (*n* = 9 for PA, none for FV). A *p*-value of .05 was defined as the level of significance.

## Results

### Participants’ characteristics

Table 1 shows the characteristics of the 346 participants in the intervention group at baseline. The mean age is 50.91 years (range 22–84) and the sample includes more females (65.2 %) than males. 46.0 % of the participants had a mid-educational level, 77.4 % of the participants were in a relationship, and 63.1 % were employed. Regarding the compliance with the behavioural guidelines, we have found that 153 (44.2 %) of the study participants were physically active for at least 30 min a day on at least 5 days a week, and that 144 (41.7 %) of the study participants ate five portions of fruits and vegetables a day.

**Table 1:** Baseline intervention group characteristics and correlations of study variables

	*N* (%)	Mean (SD)	1	2	3	4	5	6	7	8	9	10	11	12	13	14	15	16
1 Age		50.91 (12.93)																
2 Gender			-.17^a^															
Male	123 (35.5)																	
Female	223 (64.5)																	
3 Educational level			-.18^a^	.04														
High	95 (22.5)																	
Middle	158 (45.8)																	
Low	95 (27.5)																	
4 Relationship			.05	-.09	-.00													
Single	77 (22.4)																	
In relationship	267 (77.6)																	
5 Working situation			-.29^a^	.04	.21^a^	-.00												
Unemployed	126 (36.7)																	
Employed	217 (63.3)																	
6 Accomplishes PA recommendation			.10	-.19^a^	-.18^a^	.08	.06											
Yes	153 (44.2)																	
No	193 (55.8)																	
7 Accomplishes FV recommendation			.09	-.04	.03	.06	.03	.10^b^										
Yes	144 (41.7)																	
No	201 (58.3)																	
8 Risk perception		3.34 (1.26)	.07	-.09	.08	-.09	-.08	-.24^a^	-.07									
9 Outcome expectancies PA pro		6.23 (1.09)	.06	.06	.07	-.04	-.05	.02	.05	-.01								
10 Outcome expectancies PA con		3.37 (1.51)	-.20	.06	.07	-.02	.05	-.22^a^	-.21^a^	.08	-.21^a^							
11 Outcome expectancies FV pro		6.23 (1.09)	-.08	.32	.06	.02	.11	.06	.20^a^	-.17^a^	.19^a^	.31^a^						
12 Outcome expectancies FV con		3.37 (1.51)	-.25^a^	.16^a^	.05	-.16^a^	-.11	-.04	-.21^a^	.18^a^	-.02	-.30^a^	-.01					
13 Self-efficacy PA		4.67 (1.33)	.21^a^	.14^a^	-.12	.03	-.15^b^	.39^a^	.18^a^	-.15^b^	.23^a^	.00	-.25^a^	-.11				
14 Self-efficacy FV		4.73 (1.55)	.10	.08	-.09	.16^b^	.00	.20^a^	.26^a^	-.09	.03	.03	.45^a^	-.25^a^	.40^a^			
15 Intention PA intensive		3.69 (1.87)	.00	-.09	-.08	-.04	.02	.33^a^	.14^b^	-.09	.29^a^	-.26^a^	.07	-.07	.44^a^	.10		
16 Intention PA moderate		4.93 (1.60)	-.04	.15^b^	−12^b^	-.10	-.04	.00	-.05	.03	.23^a^	-.15^b^	.09	.01	.13^b^	.04	.13	
17 Intention FV		3.38 (2.14)	.13^b^	-.11	-.07	.08	-.06	.13^b^	.16^b^	-.14^b^	-.05	-.11	-.13^b^	-.07	.14^b^	.09	.04	.04

*PA* physical activity, *FV* fruit and vegetable, ^a^ = correlation is significant at 0.01 level, ^b^ = correlation is significant at 0.05 level, Adequate correlations indices were used, depending on the type of variables (i.e. Phi, Somers’d, Spearman). 1–7 is the possible range for variables 8 17

### Intervention and action planning module use

**Fig. 2:**
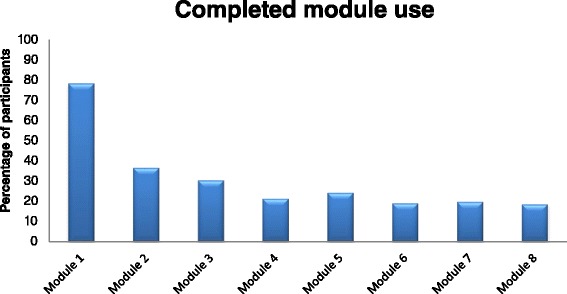
Intervention use rate

Figure 2 gives an overview about the percentage of participants from the intervention group that took part in the individual intervention module. The participation rate continuously decreased each week, beginning with 90.8 % in the first module and ending with 19.9 % in the last module.

Furthermore, data derived from the online intervention shows that 153 (44.2 %) of the participants made an AP regarding PA (module 2) and 76 (22.0 %) participants made an AP regarding FV consumption (module 6). It is possible that participants only generated actions plans, but did not complete the whole module. Hence, there is a difference between the number of generated APs and the percentage of participants who completed a module.

Predicting the intervention use, we ran a stepwise linear regression analysis. The third model shows that intervention use was significantly predicted by participants being in a relationship, having a higher risk perception, complying with the PA recommendation, and having positive intentions in terms of FV consumption (Table 2).

**Table 2:** Linear regression results: predictors of the number of completed intervention modules

	Model 1		Model 2		Model 3	
	Coefficient^a^	*P*	Coefficient^a^	*P*	Coefficient^a^	*P*
Age	.06	.34	.08	.28	.02	.68
Gender	-.09	.13	-.05	.47	-.03	.63
Relationship	.14	.02	.15	.02	.14	.02
Education	.01	.86	.05	.47	.04	.50
Working situation	-.09	.18	-.11	.10	-.08	.19
Risk perception			.07	.27	.13	.04
Recommendation PA			.20	.01	.18	.01
Recommendation FV			-.04	.51	-.08	.21
Outcome expectancies pos. PA			-.08	.23	-.03	.60
Outcome expectancies neg. PA			-.10	.19	-.06	.35
Outcome expectancies pos. FV			.15	.05	.09	.20
Outcome expectancies neg. FV			.00	.99	-.01	.95
Self-efficacy PA			.02	.77	-.01	.93
Self-efficacy FV			-.11	.16	-.10	.17
Intention FV					.46	<.001
Intention (PA intensive)					-.01	.88
Intention (PA moderate)					.06	.33
R^2^	.055		.123		.323	

^a^standardised beta

Next, we analysed the predictors of generating APs (Table 3). The third model (using all predictors of the stepwise logistic regression analysis) indicated that participants who were more physically active at baseline generated significantly less APs concerning PA.

**Table 3:** Logistic regression results: predictors of action planning

	Physical activity action plans^a^						Fruit & vegetable consumption action plans^b^					
	Model 1		Model 2		Model 3		Model 1		Model 2		Model 3	
	OR (95 % CI)	*P*	OR (95 % CI)	*P*	OR (95 % CI)	*P*	OR (95 % CI)	*P*	OR (95 % CI)	*P*	OR (95 % CI)	*P*
Age	1.00 (0.98–1.02)	.91	0.99 (0.97–1.02)	.85	1.00 (0.97–1.02)	.98	1.00 (0.97–1.02)	.82	1.00 (0.97–1.02)	.96	0.99 (0.96–1.02)	.45
Gender (ref. = female)	1.10 (0.63–1.91)	.71	0.93 (0.52–1.66)	.81	0.94 (0.52–1.70)	.84	1.31 (0.71–2.40)	.37	1.27 (0.68–2.38)	.44	0.97 (0.47–2.04)	.94
Relationship (ref. = relation)	0.73 (0.40–1.31)	.29	0.72 (0.39–1.32)	.29	0.69 (0.37–1.26)	.23	0.56 (0.26–1.20)	.13	0.50 (0.23–1.10)	.08	0.54 (0.23–1.27)	.16
Education high (ref.)		.29		.17		.17		.78		.78		.82
Low	1.04 (0.51–2.12)	.89	0.88 (0.41–1.85)	.74	0.87 (0.41–1.86)	.72	1.08 (0.49–2.37)	.83	1.27 (0.56–2.84)	.55	1.05 (0.40–2.71)	.91
Middle	0.68 (0.38–1.22)	.20	0.57 (0.31–1.07)	.08	0.56 (0.30–1.06)	.08	1.11 (0.64–2.46)	.50	1.25 (0.63–2.46)	.51	1.26 (0.57–2.81)	.57
Working situation (ref. = worker)	1.40 (0.79–2.46)	.24	1.45 (0.80–2.62)	.21	1.47 (0.81–2.67)	.20	1.48 (0.79–2.77)	.21	1.45 (0 76–2.75)	.24	1.58 (0.76–3.31)	.22
Risk perception			1.01 (1.00–1.02)	.04	1.01 (1.00–1.02)	.06			1.01 (0.99–1.02)	.07	1.02 (1.01–1.04)	.01
Recommendation			0.43 (0.23–0.80)	.01	0.46 (0.25–0.85)	.01			0.73 (0.40–1.34)	.32	0.87 (0.43–1.76)	.68
Outcome exp. pos.^c^			0.99 (0.76–1.28)	.95	0.93 (0.71–1.21)	.60			1.02 (0.76–1.37)	.86	0.81 (0.59–1.14)	.23
Outcome exp. neg.^c^			0.95 (0.76–1.28)	.65	0.97 (0.81–1.18)	.83			0.95 (0.78–1.16)	.65	0.96 (0.77–1.19)	.70
Self-efficacy^c^			1.06 (0.85–1.33)	.56	1.00 (0.79–1.27)	.99			0.86 (0.69–1.08)	.20	0.83 (0.64–1.07)	.15
Intention FV											1.79 (1.53–2.10)	<.001
Intention (PA intensive)					1.12 (0.95–1.32)	.17						
Intention (PA moderate)					1.08 (0.90–1.30)	.37						
R^2^	.032		.094		.106		.035		.062		.361	

^a^PA *n* = 260, 24.9 %missing cases; ^b^ FV *n* = 2782, 18.5 % missing cases, ^c^ Determinants specific to related behaviour

Additionally, the results of the third model in Table 3 for FV consumption indicated high risk perception and a strong intention to eat five portions of FVs daily as significant predictors of the generation of APs for this behaviour.

### Action plan quality

The majority of the APs for PA (*n* = 277, 96.9 %) and all APs regarding FV consumption (*n* = 139, 100 %) were considered instrumental (i.e. they provided a goal-directed, reasonable action). More than half of the APs for PA (69.68 %) and for FV intake (59.71 %) were found to be highly specific (providing a detailed description of the plan). Table 4 shows the results for both APs for each behaviour. Furthermore, as Table 5 shows, most plans were evaluated by the participants as useful when using the “PEPP-rule”.

**Table 4:** Action plan specificity for PA and FV consumption

	Specificity action plan PA *n* (%)		Specificity action plan FV *n* (%)	
	AP 1	AP 2	AP 1	AP 2
Not specific	3 (2.0)	7 (5.1)	3 (4.0)	5 (7.5)
Medium specific	41 (27.0)	44 (32.4)	26 (34.7)	25 (37.3)
Highly specific	108 (71.0)	85 (62.5)	46 (61.3)	37 (55.2)

**Table 5:** Results of the usefulness of the action plans with the use of the PEPP-rule

	Physical activity *n* (%)						Fruit and vegetable *n* (%)					
	Plan 1			Plan 2			Plan 1			Plan 2		
	Not useful	A bit useful	Very useful	Not useful	A bit useful	Very useful	Not useful	A bit useful	Very useful	Not useful	A bit useful	Very useful
Proper	2 (1.9)	18 (17.5)	83 (80.6)	1 (1.0)	20 (20.8)	75 (78.1)	3 (4.5)	27 (40.9)	36 (54.5)	2 (3.1)	26 (40.6)	36 (56.3)
Efficient	2 (2.0)	20 (19.6)	80 (78.4)	2 (2.1)	19 (19.8)	75 (78.1)	8 (12.1)	35 (53.0)	23 (34.8)	6 (9.4)	35 (54.7)	23 (35.9)
Practicable	6 (5.8)	35 (34.0)	62 (60.2)	7 (7.3)	36 (37.5)	53 (55.2)	2 (3.1)	32 (49.2)	31 (47.7)	4 (6.3)	28 (43.8)	32 (50.0)
Precise	7 (6.8)	39 (37.9)	57 (55.3)	11 (11.5)	30 (31.3)	55 (57.3)	6 (9.2)	21 (32.3)	38 (58.5)	5 (7.9)	18 (28.6)	40 (63.5)

A linear regression analysis (Table 6) indicated that being in a relationship is a significant predictor of AP specificity regarding PA.

**Table 6:** Linear regression results: predictors of action planning specificity

	Physical activity action plan specificity						Fruit & vegetable consumption action plan specificity					
	Model 1		Model 2		Model 3		Model 1		Model 2		Model 3	
	Coefficient^b^	*P*	Coefficient^b^	*P*	Coefficient^b^	*P*	Coefficient^b^	*P*	Coefficient^b^	*P*	Coefficient^b^	*P*
Age	.10	.27	.07	.48	.07	.48	-.02	.90	-.08	.58	-.08	.62
Gender	-.05	.53	-.02	.79	-.01	.94	.21	.11	.17	.21	.17	.22
Relationship	.29	.001	.28	.001	.28	.002	.19	.12	.15	.26	.16	.26
Education	-.11	.21	-.09	.32	-.10	.37	.10	.57	.11	.43	.11	.42
Working situation	-.04	.63	-.03	.71	-.04	.68	.05	.71	.01	.94	.01	.92
Risk perception			.09	.32	.12	.37			-.08	.54	-.08	.52
Recommendation			.10	.27	.09	.35			-.84	.41	-.11	.42
Outcome exp. pos.^a^			.01	.85	.09	.84			-.09	.93	-.01	.98
Outcome exp. neg.^a^			.01	.95	.02	.98			−1.33	.19	-.18	.19
Self-efficacy ^a^			.14	.15	.14	.15			.14	.41	.14	.42
Intention FV											-.04	.77
Intention (PA intensive)					-.06	.56						
Intention (PA moderate)					.03	.78						
R^2^	.134		.172		.176		.093		.138		.139	

^a^Determinants specific to related behaviour, ^b^standardised beta

## Discussion

### Main findings

#### Use of intervention

The purpose of this study was to evaluate the usage, generation and quality of APs concerning physical activity and fruit and vegetable consumption within a web-based computer tailored intervention. This study shows that the intervention modules are used scarcely, that less than half of the participants generated APs, and that the quality of these APs was high. Another study that made use of action planning modules by which participants were required to generate their own APs also reported a low level of participation [46]. A known shortcoming of web-based intervention is the low level of usage [47]. The reason for “non-usage attrition” can vary in terms of interventional factors, demographic characteristics [48], and social-cognitive causes [49–51]. With regards to demographic characteristics, we only found that participants who were in a relationship made more use of the intervention. While people who are in a relationship have typically healthier lifestyles [52] and report a better perceived health [53], it is also known that individuals in a relationship are less physically active and have a higher body weight than single people [54]. This could explain why people who are in a relationship have a high interest in using the intervention to improve their PA.

Furthermore, we found that participants who have a high risk perception with regards to cardiovascular diseases and those who have a strong intention to change their FV consumption behaviour made more use of the intervention modules. This corresponds with the findings that risk perception [55] and intention [56] are positively related to a change in health behaviour. People with a high level of risk perception tend also to seek more information [57]. This might suggest that those participants who have high risk perception used the intervention to gain more information on how to change their health behaviours, which might also be true for highly motivated participants.

Participants at baseline who complied with the PA recommendation made more use of the intervention and this may be due to the positive feedback that they received during the intervention due to their behaviour. Participants who complied with the behavioural recommendation at the start of the intervention got positive feedback and received less information about how to change their behaviour. For those participants, the intervention might be less time-consuming in contrast to participants who did not comply with the recommendation. They received more feedback and were asked to change their behaviour. If people need to process a lot of new information, it requires a lot of resources and might decrease their self-regulatory capacity which is called ego depletion [58, 59] which could hinder participants from participating in all single intervention modules.

### Generation of action plans

Those participants who were more physically active at baseline were less likely to generate APs for PA. It might be that participants who were already active skipped the application of the AP because they have more experiences about when, where, and how to be physically active. Furthermore, it is reasonable that less physically active participants made more use of the AP modules because they received more tailored feedback on their behaviour and how to generate APs, which might have encouraged them to use the application more.

We have found that participants who had a high risk perception generated more APs for FV consumption. As mentioned above, those participants might be more interested in relevant information about how to change their health behaviour [57] and might therefore generate APs.

Furthermore, having a strong intention to increase one’s FV consumption was found to be a significant predictor of generating APs for that behaviour. Since intention is one of the most important determinants of behavioural change [56] and a parameter of use with regards to optimising the effectiveness of APs [60], it seems plausible that participants who had a strong intention to change made the effort to generate APs to increase their self-regulation skills in terms of performing this behaviour in practice [61].

### Action plan quality

We found that nearly all APs were highly instrumental, indicating that they were goal-directed. It is reasonable to assume that only participants who had a high intention to change their behaviours used this intervention module and made serious efforts to generate APs. In addition, more than half of the plans were highly specific and provided a high amount of details about what, where, when, how, and with whom the plan will be performed. APs that were medium-specific did not mention all details of the defined AP. This is in line with another study about the quality of action planning that reported that less specific plans did not mention the time at which the action would be performed [46]. Nevertheless, the good quality of the plans could be explained by the fact that participants were guided through the planning modules by providing examples of effective plans, by asking participants to fill in their plans step-by-step, and because participants needed to evaluate their APs using the “PEPP-rule”. This practical application seems to be useful in terms of generating high quality APs.

Regarding predictors of plan specificity, we found that being in a relationship has a positive influence on the quality of APs. It is conceivable that this is biased with the presence of the partners while generating the AP and participants filled in the “with whom” section with their partner, which resulted in a higher plan specificity as defined.

Contrary to our assumption that participants that have different socio-demographic characteristics might use the intervention modules differently [48] or might generate different plans with regards to plan quality, no such difference could be found. This might imply that the “PEPP-rule” application is equally effective for people who have different educational levels or different working situations to generate instrumental and specific APs.

### Strengths, limitations, and recommendations

One of the strengths of the study is that we were able demonstrate in our web-based tailored computer intervention that not only did participants generate APs but that they were generally of high quality. We distinguished between instrumentality and specificity. AP quality had found little attention in the existent literature. This study adds to previous research that the “PEPP-rule” can provide a valuable means to generate instrumental and specific APs in an eHealth intervention. The results of this study provide further insight as to how usage of intervention modules depends on socio-demographic variables and social cognitive variables. A further strength of our results is that the generated APs were part of an intervention and were not gained from a clinical setting. This might allow generalisation of the results to the field of web-based interventions, thereby expanding the insights into previous studies that were carried out in more controlled settings.

There are also limitations of our study. The first limitation is that intervention use decreased rapidly from module to module and that only a minority of participants generated APs regarding FV consumption. This could be caused by the given sequence of the intervention. Participants had no choice as to which module they preferred to improve and neither did participants have the opportunity to choose with which behaviour they preferred to start. Therefore, it could be possible that participants who were interested in changing their FV consumption did not participate in certain intervention modules because they had no interest in completing the modules about PA. It is not yet known whether it is advisable to give participants the control to choose what to do within an intervention or to guide participants through an intervention. Participants who were guided through a website and had less control about what to do remembered more information afterwards [62], while a study found that participants who had no choices in an online intervention dropped out more often [63]. Further studies might want to find a balance between guiding participants through an intervention while also providing individual choices.

Secondly, we did not find any predictors for AP specificity for FV consumption. This could have been caused by the small amount of participants that generated APs for this behaviour.

Finally, within the study, we tried to optimise the scope of the intervention by recruiting participants via different channels such as advertisements in hospitals, in newspapers, and on social media networks; and this study might, therefore, be vulnerable to selection bias [34]. We assumed that mainly people with a strong intention to change their health behaviour would register themselves for participation. Therefore, our intervention was mainly focused on participants within the volitional phase of behaviour change. For participants who did not develop the intention to change their behaviour, the intervention was perhaps less suitable. The focus on action planning can only result in behavioural change if participants have a positive intention. Further interventions should add modules for participants in the motivational phase to prevent non-usage attrition [8].

## Conclusion

While our intervention was scarcely used, the generated APs were of a high quality. The generated APs were highly instrumental and specific. Providing a guided step-by step application with the opportunity to adjust APs by using the “PEPP-rule” seems to be a promising application to formulate APs for highly motivated participants who wish to improve their PA and FV consumption.

### Avaliability of data and materials

The data which was used in the current study is available and can be obtained from the authors. We have uploaded the dataset on Open Science Framework.

## Abbreviations

AP: action plan
FV: fruit and vegetable
HAPA: health action process approach
PA: physical activity
